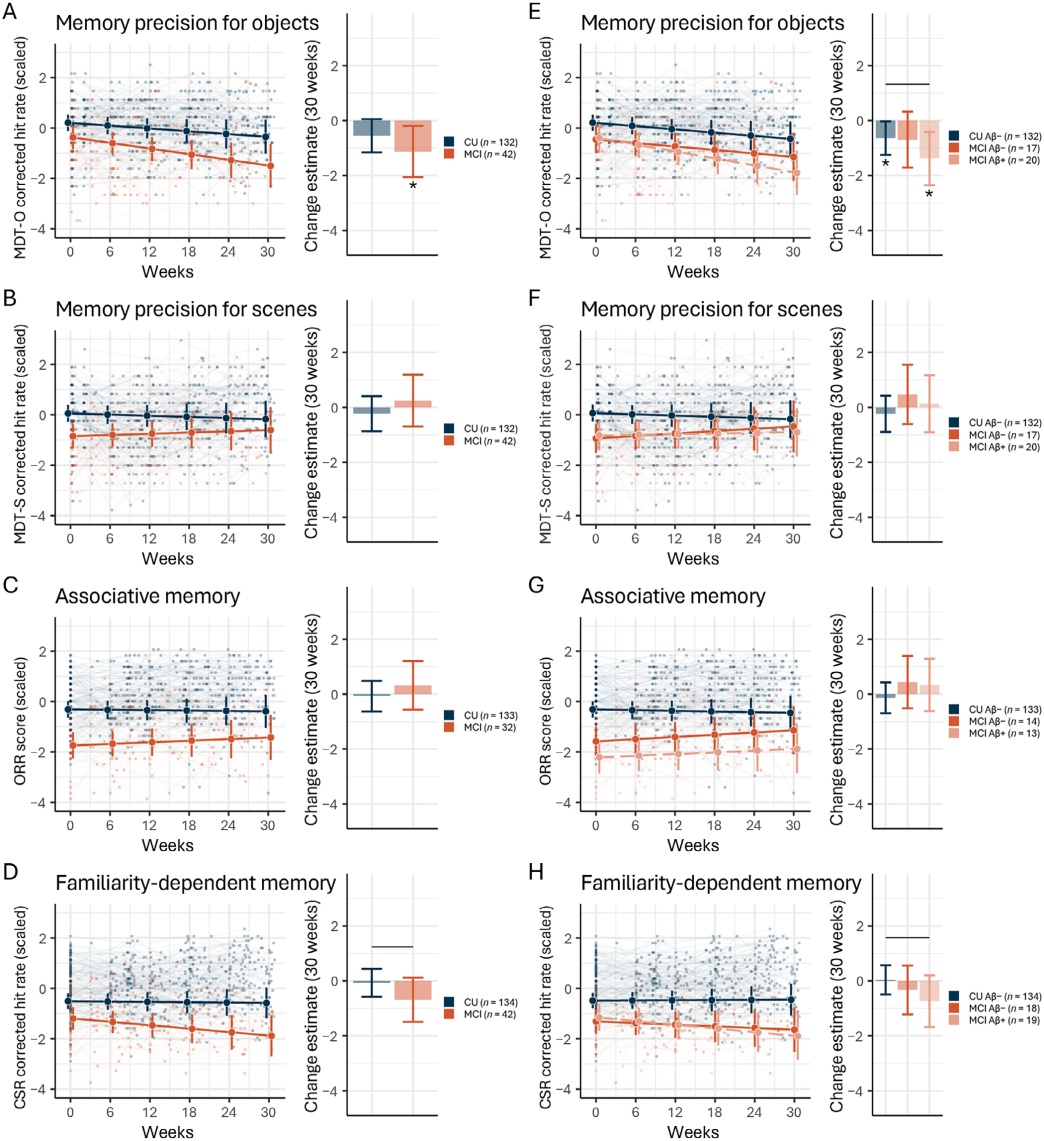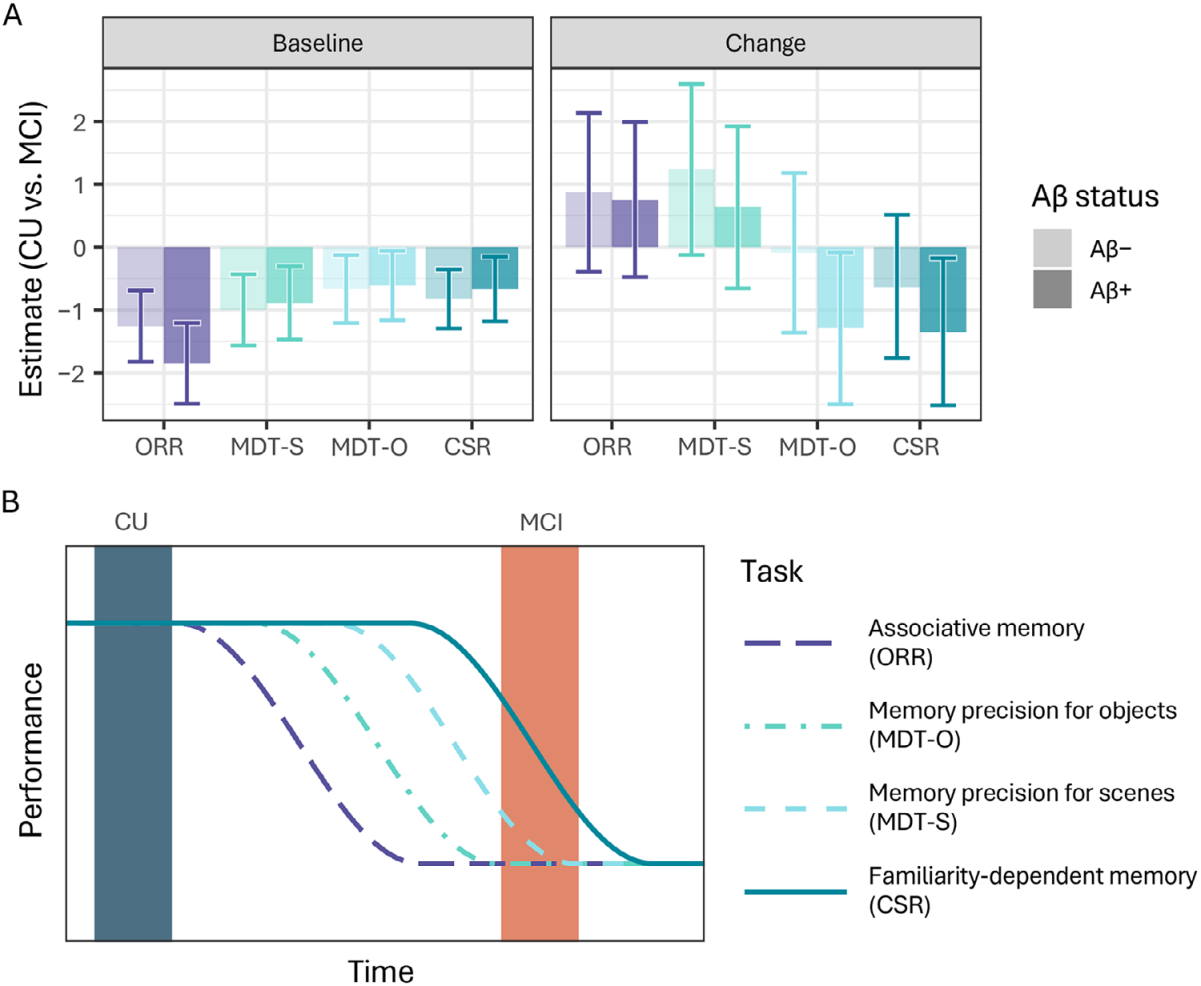# Remote and unsupervised monitoring of episodic memory decline in patients with prodromal Alzheimer's disease

**DOI:** 10.1002/alz70863_110650

**Published:** 2025-12-23

**Authors:** Sarah E. Polk, Lindsay R Clark, Kristin E Basche, Luca Kleineidam, Wenzel Glanz, Michaela Butryn, Robert Perneczky, Katharina Buerger, Klaus Fliessbach, Christoph Laske, Annika Spottke, Anja Schneider, Jens Wiltfang, Stefan J. Teipel, Michael Wagner, Sterling C Johnson, Frank Jessen, Emrah Düzel, David Berron

**Affiliations:** ^1^ German Center for Neurodegenerative Diseases (DZNE), Magdeburg Germany; ^2^ Division of Geriatrics, Department of Medicine, University of Wisconsin School of Medicine and Public Health, Madison, WI USA; ^3^ Wisconsin Alzheimer's Disease Research Center, University of Wisconsin‐Madison School of Medicine and Public Health, Madison, WI USA; ^4^ Geriatric Research Education and Clinical Center, William S. Middleton Memorial Veterans Hospital, Madison, WI USA; ^5^ Department of Old Age Psychiatry and Cognitive Disorders, University Hospital Bonn and University of Bonn, Bonn Germany; ^6^ German Center for Neurodegenerative Diseases (DZNE), Bonn Germany; ^7^ Ageing Epidemiology (AGE) Research Unit, School of Public Health, Imperial College London, London UK; ^8^ Munich Cluster for Systems Neurology (SyNergy), Munich Germany; ^9^ German Center for Neurodegenerative Diseases (DZNE), Munich Germany; ^10^ Department of Psychiatry and Psychotherapy, University Hospital, LMU Munich, Munich Germany; ^11^ Institute for Stroke and Dementia Research (ISD), University Hospital, LMU, Munich Germany; ^12^ German Center for Neurodegenerative Diseases (DZNE), Tübingen Germany; ^13^ Section for Dementia Research, Hertie Institute for Clinical Brain Research and Department of Psychiatry and Psychotherapy, University of Tübingen, Tübingen Germany; ^14^ Department of Neurology, University of Bonn, Bonn Germany; ^15^ German Center for Neurodegenerative Diseases (DZNE), Göttingen Germany; ^16^ Department of Psychiatry and Psychotherapy, University Medical Center Goettingen, University of Goettingen, Goettingen Germany; ^17^ Neurosciences and Signaling Group, Institute of Biomedicine (iBiMED), Department of Medical Sciences, University of Aveiro, Aveiro Portugal; ^18^ Department of Psychosomatic Medicine, Rostock University Medical Center, Rostock Germany; ^19^ German Center for Neurodegenerative Diseases (DZNE), Rostock Germany; ^20^ Department of Medicine, University of Wisconsin‐Madison School of Medicine and Public Health, Madison, WI USA; ^21^ Excellence Cluster on Cellular Stress Responses in Aging‐Associated Diseases (CECAD), University of Cologne, Cologne Germany; ^22^ Department of Psychiatry, Medical Faculty, University of Cologne, Cologne Germany; ^23^ Institute of Cognitive Neurology and Dementia Research (IKND), Otto‐von‐Guericke University, Magdeburg, Sachsen Anhalt Germany; ^24^ Center for Behavioral Brain Sciences (CBBS), Otto‐von‐Guericke University Magdeburg, Magdeburg, Sachsen Anhalt Germany; ^25^ Clinical Memory Research Unit, Department of Clinical Sciences, Lund University, Lund Sweden

## Abstract

**Background:**

Traditional pen‐and‐paper neuropsychological assessments are not sensitive to subtle cognitive changes in the earliest stages of Alzheimer's disease (AD), limiting their use for monitoring of cognitive performance over shorter timeframes. Here, we show that frequently administered remote and unsupervised digital cognitive assessments are better suited to capture short‐term cognitive decline in early AD.

**Method:**

We investigated episodic memory trajectories using self‐administered remote digital testing in 202 participants (52–85 years) who completed unsupervised tests for at least 30 weeks. Linear mixed modeling was used to investigate main effects of cognitive status, *n* =  152 cognitively unimpaired (CU), *n* =  50 with mild cognitive impairment (MCI), and interaction effects of cognitive status by time spent in the study. Analyses were repeated, stratifying the MCI group by amyloid‐β (Aβ) burden (*n*_Aβ−_ = 21, *n*_Aβ+_ = 24). Baseline and change‐change associations with in‐person neuropsychological assessments were also examined using Pearson correlations.

**Result:**

At baseline, MCI performed worse than CU on an associative memory task (Object‐in‐Room Recall, ORR), memory precision tasks for objects and scenes (Mnemonic Discrimination Task for Objects and Scenes, MDT‐OS), and a familiarity‐based memory task (Complex Scene Recognition, CSR). A short‐term decline in the familiarity‐dependent task was observed in all patients with an MCI diagnosis, while both the familiarity‐dependent memory task and memory precision for objects task were sensitive to decline in the MCI Aβ+ group specifically. Change in the remotely assessed familiarity‐dependent memory was correlated with multi‐year change on annual in‐person neuropsychological assessments. Finally, in‐person tests were not sensitive to short‐term cognitive changes in MCI.

**Conclusion:**

Altogether, these findings show that frequent remote cognitive testing is a promising tool to feasibly capture subtle and short‐term cognitive decline.